# Impact of left ventricular hypertrophy on frequency and complexity of ventricular arrhythmia among hypertensive Egyptian patients

**DOI:** 10.1186/s43044-024-00472-8

**Published:** 2024-03-26

**Authors:** Ahmed Mokhtar Abd El Azeem, Mohamed Ahmed Abd Elmoneim, Samir Morkos Rafla, Gehan Magdy Youssif

**Affiliations:** 1 Cardiology Department, Faculty of Medicine, Alexandria University, Champlion Street, El Azarita, Alexandria, Egypt; 2 Kafr El-Dawar General Hospital, Ministry of Health, Kafr El Dawwar, Egypt

**Keywords:** Left ventricular hypertrophy, Left ventricular mass index, Ventricular premature contraction, Ventricular arrhythmia, Lown grading

## Abstract

**Background:**

Left ventricular hypertrophy (LVH) is associated with an increased risk of ventricular arrhythmias and cardiovascular mortality. The study objective was to investigate the effect of LVH severity on the complexity and severity of ventricular arrhythmias among a subset of Egyptian hypertensive patients.

**Results:**

The study cohort consisted of 60 hypertensive patients with LVH as diagnosed by echocardiography. Their mean age was (53.7 ± 12.3) years. 36 males (40%) and 24 females (60%). Diabetes mellitus was found in 26 patients (43%), 41% of these cases were smokers. 48-h Holter monitoring was performed in all cases to assess the frequency of ventricular premature contractions (VPC) and their complexity using the Lown grading. Increasing posterior wall thickness end diastole (PWTd) was an independent predictor of increasing VPC frequency, each 1 mm increase in the PWTd is associated with a 1.26% increase in the VPC% among total heart beats (*b* = 12.6, *p* < 0.001). Higher-grade VPCs—defined as grade 4a, 4b, and 5—were seen in 29 patients (48.3%). Interventricular septum thickness end diastole, PWTd, left ventricular mass, and left ventricular mass index (LVMI), were significantly higher among patients with higher Lown grading (*p* < 0.001). Using logistic regression analysis, female sex and LVMI were independent predictors of more complex VPC (OR = 8.766, *p* = .014), (OR = 1.096, *p* < 0.001), respectively. Among females, LVMI of more than 120 g/m^2^ can differentiate between high- and low-grade VPCs with 71% sensitivity and 80% specificity, while among males, LVMI of more than 129.5 g/m^2^ can differentiate between high and low-grade VPCs with 86% sensitivity and 66% specificity.

**Conclusions:**

The frequency and complexity of ventricular arrhythmias among hypertensive heart disease are correlated with the severity of ventricular hypertrophy. Female sex and increasing left ventricular mass index are independent predictors of more complex forms of ventricular arrhythmias.

## Background

Many studies have examined the relationship between left ventricular hypertrophy (LVH), ventricular arrhythmia (VA), and sudden cardiac death (SCD) since the initial report from the Framingham cohort [1] addressed LVH as a strong independent predictor of cardiovascular morbidity and mortality. Several [2–7] but not all [8–10] prior investigations have shown an elevated incidence of ventricular tachyarrhythmias among individuals with LVH detected by either echocardiography or electrocardiography. Nevertheless, it is unclear if elevated left ventricular mass (LVM) as a result of hypertension poses a separate risk for ventricular tachyarrhythmias. There are additional factors that contribute to the increased risk of arrhythmogenesis, such as concomitant coronary artery disease (CAD), associated ventricular systolic dysfunction, and electrolyte abnormalities [11]. We, therefore, investigated the occurrence of ventricular arrhythmias and their complexity in a group of hypertensive Egyptian patients—in whom epicardial coronary artery disease was ruled out by invasive coronary angiography—and the degree of LVH as assessed by echocardiographic evaluation.

The purpose of the study was to evaluate the impact of increasing left ventricular wall thickness and mass on the complexity and severity of ventricular arrhythmias among these subsets of hypertensive patients without concomitant CAD or left ventricular systolic dysfunction.

## Methods

### Study patients

Sixty hypertensive individuals with confirmed left ventricular hypertrophy (LVH) on echocardiography constituted the study cohort. If a patient's blood pressure was measured at more than 140/90 mm Hg on one or more outpatient visits, had a history of hypertension, or was started on antihypertensive medication, they were diagnosed with hypertension. The patients' recommended anti-hypertensive medications remained unchanged. No sedatives, antipsychotics, or antiarrhythmics (apart from beta-adrenergic blockers) were administered to the patient. Informed consent was given by the patients. For men, a left ventricular mass index (LVMI) of greater than 115 g/m^2^ and for women, greater than 95 g/m^2^ was considered left ventricular hypertrophy.

### Exclusion criteria

In cases where technically appropriate echocardiographic images could not be acquired, patients were excluded from the study protocol. Individuals having a history of atrial fibrillation or CAD were not accepted. To rule out any concomitant arrhythmogenic conditions, patients were excluded if their echocardiogram revealed asymmetric septal hypertrophy (septal to posterior free wall thickness ratio > 1.3), left ventricular dilation (diastolic left ventricular diameter > 5.8 cm), impaired ventricular systolic function (ejection fraction (EF) less than 50%), or significant valve disease.

### Echocardiographic assessment

A qualified cardiologist evaluated each patient's echocardiogram using the standard views—parasternal, short-axis, and apical—while positioning them in the left lateral position, in compliance with the American Society of echocardiogram guidelines [12]. Three heartbeats were averaged for the assessment. The following measurements were made of the left ventricle's internal diameter in systole (LVIDS) and diastole (LVIDD), as well as the interventricular septal thickness at end diastole (IVSTd) and posterior wall thickness at end diastole (PWTd).

LVM was computed utilizing the modified Devereux formula [13]:

$${\text{LVM}} = 0.{8 }\left[ {{1}.0{4}\;\left( {{\text{IVSTd}} + {\text{LVIDD}} + {\text{PWTd}}} \right)^{{3}} - \left( {{\text{LVIDD}}} \right)^{{3}} } \right] + 0.{6}\;{\text{g}}.$$

Left ventricular mass index (LVMI) was calculated after dividing LVM by body surface area (LVM/BSA, g/m^2^).

The formula used to compute the body surface area (in square meters) was 0.0001 × 7l.84 (weight in kg)^0.425^ × (height in cm) ^0.725^ [14].

### Ambulatory ECG monitoring

Cardio UP three-channel recorders were used to capture all ambulatory ECG tracings throughout 48 monitoring hours. Each tape was scanned and reviewed for detailed analysis by two cardiologists who did not know the results of other investigations.

### Ventricular arrhythmias were categorized according to the following

The frequency of premature ventricular beats (VPB) as a percentage of the total heart rate per 24 h in Holter monitoring and the complexity of ventricular premature beats according to Lown and Wolf [15].

0 No VPB

1 Occasional, isolated VPB

2 Frequently occurring VPBs; more than 1 per minute or more than 30 betas per hour

3 Multiform VPB

4(a) Repetitive VPB: couplets

4(b) Repetitive VPB: salvos

5 Early VPB

We further classified them into low grades (Lown grades 1,2 and 3) and high grades (Lown grades 4a, 4b, and 5).

### Statistical analysis

The Statistical Package for Social Sciences (SPSS ver.28 Chicago, IL, USA) was used to analyze the data. Data were presented using mean and standard deviation (SD), and the K-S test was used to test for normality. Frequency and percentage descriptions were provided for the qualitative variables. The high and low Lown grade groups were compared using an independent sample t-test. The diagnostic accuracy of LVMI was tested using ROC curve analysis, and the cutoff point with the best sensitivity and specificity was identified. The prediction of increasing VPC complexity (Grades 4a, 4b, 5 vs. grades 1, 2, 3) was made using multivariate logistic regression analysis. Predictors were included in the regression models using the enter technique, and significant contributing variables were identified by calculating the adjusted odds ratio with a 95% confidence interval. The percentage of VPCs in total HR was predicted using multiple linear regression analysis, along with the computation of beta coefficients and t-tests. There was no multicollinearity among the predictors when the assumption of multicollinearity was verified using VIF tolerance. A p-value of 0.05 was considered statistically significant.

## Results

Our study was conducted on 60 hypertensive patients, their mean age was (53.7 ± 12.3) years, 36 males (40%) and 24 females (60%). Diabetes mellitus was found in 26 patients (43%), 41% of these cases were smokers. Regarding the grade of arrhythmia, 31 patients (51.7%) were in the low-grade VPC category (1, 2, and 3), while 29 patients (48.3%) were in the high-grade category (4a, 4b, and 5).

Comparing both groups according to risk factors and echocardiographic parameters, we found that increasing posterior wall thickness, interventricular septal thickness, and increasing LV mass and LV mass index were statistically significant for more complex ventricular arrhythmias as shown in Table 1, (*p* < 0.001).

**Table 1:** Comparison between high- and low-grade VPC according to demographic and echocardiographic parameters

	Low-grade VPC		High-grade VPC		*P* value
	Mean	(SD)	Mean	(SD)	
Age in years	52	9	55	15	0.16
Weight in kg	86	8	87	9	0.89
BSA/m^2^	1.97	0.13	1.99	0.14	0.56
LVIDd in cm	4.5	0.2	4.5	0.5	0.73
IVSTd in cm	1.4	0.1	1.6	0.2	< 0.001*
PWTd in cm	1.39	0.11	1.56	0.21	< 0.001*
LVM gm	246.18	33.5	300.90	67.4	< 0.001*
LVMI gm/m^2^	124.6	13	150.1	29.3	< 0.001*

*Statistically significant by independent sample *t*-test

To identify independent predictors of higher-grade VPC, multivariate logistic regression analysis was done, it was statistically significant (Model X^2^ = 28.8, *p* < 0.001). The model explains 49.9% of the variability in the outcome (Grade IV) (Nagelkerke R Square = 0.499). The significant predictors were: Female gender and LVMI. Females were 8.7 times more likely to develop Grade IV or more complex ventricular arrhythmias than males (OR = 8.766, *p* = 0.014). Each 1 gm/m^2^ increase in LVMI increased the odds of developing higher-grade VPC by 9.6% (OR = 1.096, *p* < 0.001). While age, weight, and LVIDD were statistically not significant predictors (Table 2).

**Table 2:** Multivariate logistic regression model predicting high-grade VPC

	*B*	S.E	*p*	OR	95% C.I. for OR	
					Lower	Upper
Age	0.071	0.041	0.083	1.073	0.991	1.162
Gender (Female)	2.171	0.886	0.014*	8.766	1.543	49.794
Weight	− 0.052	0.041	0.201	0.949	0.876	1.028
LVMI	0.092	0.026	< 0.001*	1.096	1.041	1.154
LVIDd	− 1.872	1.074	0.081	0.154	0.019	1.263

Model X^2^ = 28.8, *p* < 0.001, Nagelkerke R Square = 0.499

* Statistically significant

*OR* odds ratio

To identify the independent predictors of the increasing daily burden of ventricular arrhythmia, we made a multiple linear regression model for the prediction of the percentage of VPBs/total heart rate, the model was statistically significant (*F* = 5.8, *p* < 0.001). The model explains 35% of the outcome (*R*^2^ = 0.35). The only statistically significant predictor was posterior wall thickness. With each 1 mm increase in PWTd, the VPC percentage increases by 1.266% of the total heart rate (*b* = 12.6, *p* < 0.001). While adjusting for other predictors (age, gender, LVMI) which were not statistically significant as shown in Table 3

**Table 3:** Multiple linear regression predicting the percentage of VPC/Total heart rate

			*t*	*p*	95% CI for B	
	B	SE			Lower bound	Upper bound
(Constant)	− 3.623	3.195	− 1.134	0.262	− 10.028	2.783
Age	0.017	0.026	0.645	0.522	− 0.036−	0.070
gender	− 0.028	0.624	− 0.045	0.964	− 1.279	1.222
Weight	− 0.112	0.042	− 2.703	0.009*	− 0.196	− 0.029−
LVMI	− 0.028	0.019	− 1.451	0.153	− 0.066	0.011
PWTd in cm	12.662	2.933	4.317	< 0.001*	6.782	18.542

*F* = 5.8, *p* < 0.001, R square = 0.35

*Statistically significant: *B* beta coefficient, *SE* standard error, *CI* confidence interval.

**Fig. 1:**
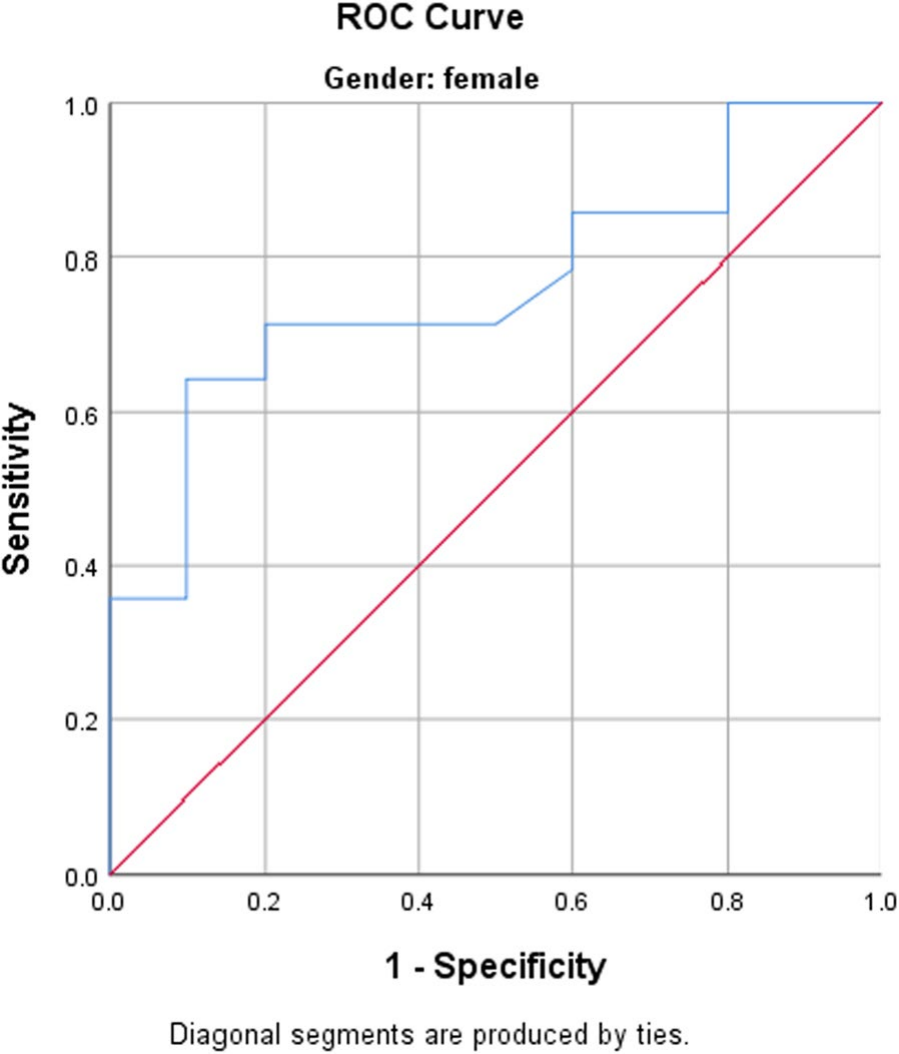
ROC curve analysis for LVMI in discriminating between low-grade and high-grade VPC in females

The accuracy of LVMI in discriminating between higher- and lower-VPC grades was studied for males and females. Among females, Using ROC curve analysis there's statistically significant accuracy of LVMI (AUC = 0.761, *p* = 0.033) in discriminating between high- and low-grade VPC (cutoff point: 120, Sensitivity = 71%, Specificity = 80%) as shown in Fig. 1.

**Fig. 2:**
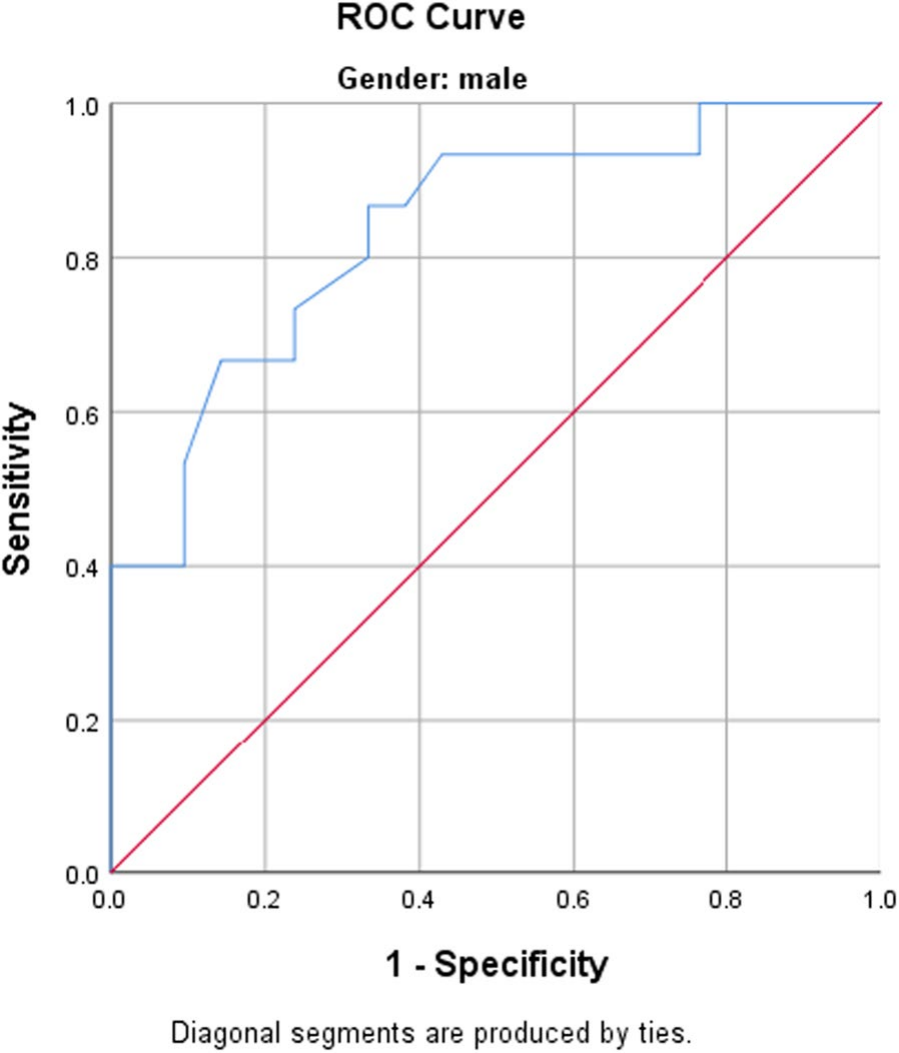
ROC curve analysis for LVMI in discriminating between high-grade and low-grade VPB in males

Among males, using ROC curve analysis, there is statistically significant accuracy of LVMI (AUC = 0.837, *p* = 0.001) in discriminating between high-grade and low-grade VPC (Cutoff point: 129.5, sensitivity = 86%, specificity = 66%) (Fig. 2).

## Discussion

In the current study, we concluded that the increased posterior wall thickness among hypertensive patients is an independent predictor of increased VPB frequency. Moreover, the more elevated LVMI and the female gender were found to be strong independent predictors of developing more complex ventricular tachyarrhythmias in the form of couplets, salvos, and R on T VPBs. Our study detected that LVMI of more than 120 g/m^2^ in females and more than 129.5 g/m^2^ in males is a strong significant independent predictor of developing more complex forms of ventricular tachyarrhythmias among our study population. Therefore, we concluded that there is a sex-specific association between left ventricular hypertrophy and the complexity of ventricular arrhythmias.

Hypertensive left ventricular hypertrophy has been associated with an increased incidence of atrial and ventricular tachyarrhythmias and consequently increased risk of cardiovascular death. This concept has been extensively studied before. However, the data on the correlation between the severity of left ventricular hypertrophy and the frequency and complexity of ventricular arrhythmia is limited. Therefore, we decided to conduct a study to fill this gap in the literature.

Our study was designed to investigate the frequency and the complexity of ventricular arrhythmias using 48 h of ambulatory ECG monitoring among hypertensive patients with left ventricular hypertrophy diagnosed by the echocardiographic criteria including the measurement of (IVSTd), (PWTd) and the (LVMI) calculated according to the Devereux’s adjusted formula. Patients with documented coronary artery disease, reduced systolic function, significant valvular disease, and patients having asymmetrical septal hypertrophy were excluded.

We found that the more severe the increase in the left ventricular hypertrophy, the more frequent the incidence of ventricular arrhythmias, where for every 1 mm increase in the PWTd, the percentage of VPCs increases by 1.266% of the total heart rate. Moreover, we detected that hypertensive females having LVH were 8.7 times more likely to develop more complex ventricular arrhythmias than hypertensive males with LVH. In addition to the female gender as an independent predictor of high-grade complex VPCs, our study concluded that LVMI is also to be considered a significant predictor; where each 1 gm/m^2^ increase in LVMI increases the odds of developing higher-grade VPCs by 9.6%.

Some of the literature has discussed the relationship between the severity of left ventricular hypertrophy and the prevalence of ventricular arrhythmias among hypertensive patients. In agreement with our results, a study conducted by Kunisek et al. [16] on 192 patients with hypertensive heart disease without coronary artery disease concluded that ventricular arrhythmias were more prevalent among those with moderate and severe LVH. Also, a large metanalysis that was carried out by Chatterjee et al. [17] suggested that the frequency of ventricular arrhythmias in patients with LVH was 5.5% compared to 1.1% in patients without LVH and that the incidence of ventricular arrhythmias was 2.8 times more common among patients with LVH. Moreover, our results were consistent with the previous studies which suggest a positive correlation between LVMI and the complexity of ventricular arrhythmias. Ozdemir et al. [18] detected that 80% of hypertensive LVH patients developed > Lown grade 2 ventricular arrhythmias in comparison to 10% in patients with no LVH. In addition to this, our study suggested that there is a positive link between increasing the severity of LVMI and increasing the incidence of developing more complex ventricular arrhythmias which has not been widely discussed in the literature. In contrast to the study conducted by Kunisek et al. [16] in 2008 which detected that the incidence of simple and complex ventricular arrhythmias was not statistically significant when correlated to the severity of LVH (*p* = 0.757 for simple versus *p* = 0.657, *p* = 0.819, *p* = 0.617, for polytopic, pairs and ventricular tachycardia, respectively, for complex ventricular arrhythmias). This conflict may be due to the small sample size and the shorter duration of monitoring. Therefore, we believe that further studies with larger study populations and longer monitoring periods are necessary to validate our results.

In our study, females represented 40% of the sample size and we found female gender an independent predictor of more complex forms of ventricular arrhythmias. To our knowledge, very scanty data are available regarding gender differences in arrhythmia occurrence among hypertensive left ventricular hypertrophy. An experimental protocol carried out by Biagetti et al. in 2006 on rabbits to study sex-related electrical remodeling and vulnerability to ventricular tachyarrhythmias due to LVH concluded that male rabbits with LVH had longer ventricular action potential durations, greater transmural repolarization dispersion, and consequently, higher incidence of ventricular arrhythmias than female rabbits with LVH [19]. By the results of this animal model-based study, Framingham [20], Moss et al. [21], and Dittrich et al. [22] concluded that the incidence of ventricular arrhythmias in the setting of coronary artery disease and consequently fatal arrhythmic events are more common among males than females. We believe this conflict could be because most of our knowledge regarding electrophysiological differences between males and females is derived from animal models and -as suggested by the ESC consensus document published in 2018- more women are needed to be enrolled in the trials to make firm conclusions regarding the sex-related differences in the electrophysiological properties and arrhythmia incidence [23]. Moreover, very little attention in the literature and almost none has been drawn to study gender-related differences in the incidence of ventricular arrhythmias in LVH due to hypertension in the absence of co-existing coronary artery disease which we believe needs further larger studies to confirm and endorse our findings.

## Study limitations

Our study had some limitations; the most important was the small sample size. The 48-h Holter monitoring duration to detect the incidence, frequency, and complexity of ventricular arrhythmias among our study population is relatively short. We believe that more frequent and more complex ventricular arrhythmias could be expected with longer monitoring duration. In addition, we depended on the ECG and the echocardiographic criteria for the detection of the LVH and the assessment of the LVMI, despite being the most widely used tools in routine practice, we think that CMR would be more helpful in the accurate assessment of the LV mass. Moreover, it provides a more comprehensive morphological characterization of the myocardium which aids in confirming the precise etiology of the LVH and it evaluates the presence and the extent of fibrosis and scarring which allows for a better understanding of the possible underlying structural substrates for ventricular arrhythmia. However, due to the higher costs of the CMR and the fear of the possible hazards such as contrast media reaction, we decided to depend in our study on low-cost, non-invasive, time-efficient, and easily reproducible tools.

## Conclusions

Left ventricular hypertrophy in hypertensive heart disease is associated with more frequent and more complex ventricular arrhythmia. There is a linear relationship between the severity of LVH and the frequency of ventricular arrhythmias. Female sex and increasing LVMI are independent predictors of developing more complex ventricular arrhythmias among hypertensive patients.

## Data Availability

The datasets used and/or analyzed during the current study are available from the corresponding author on reasonable request.

## Abbreviations

LVH: Left ventricular hypertrophy
VPC: Ventricular premature contractions
PWTd: Posterior wall thickness at end diastole
VA: Ventricular arrhythmia
SCD: Sudden cardiac death
CAD: Coronary artery disease
IVSTd: Inter ventricular septal thickness at end diastole
LVIDD: Left ventricular internal diameter in diastole
LVIDS: Left ventricular internal diameter in systole
LVMI: Left ventricular mass index
BSA: Body surface area
VPB: Premature ventricular beats
